# The Impact of Multidisciplinary Conferences on Healthcare Utilization in Chronic Pain Patients

**DOI:** 10.3389/fpain.2021.775210

**Published:** 2021-11-22

**Authors:** Zahabiya Campwala, Gregory Davis, Olga Khazen, Rachel Trowbridge, Melisande Nabage, Rohan Bagchi, Charles Argoff, Julie G. Pilitsis

**Affiliations:** ^1^Department of Neuroscience and Experimental Therapeutics, Albany Medical College, Albany, NY, United States; ^2^Department of Neurosurgery, Albany Medical Center, Albany, NY, United States; ^3^Department of Neurology, Albany Medical Center, Albany, NY, United States

**Keywords:** chronic pain, multidisciplinary conference, multidisciplinary team, healthcare utilization, clinic visits

## Abstract

Approximately 100 million adults in the United States have chronic pain, though only a subset utilizes the vast majority of healthcare resources. Multidisciplinary care has been shown to improve outcomes in a variety of clinical conditions. There is concern that multidisciplinary care of chronic pain patients may overwhelm existing resources and increase healthcare utilization due to the volume of patients and the complexity of care. We report our findings on the use of multidisciplinary conferences (MDC) to facilitate care for the most complex patients seen at our tertiary center. Thirty-two of nearly 2,000 patients seen per year were discussed at the MDC, making up the top 2% of complex patients in our practice. We evaluated patients' numeric rating score (NRS) of pain, medication use, hospitalizations, emergency department visits, and visits to pain specialists prior to their enrollment in MDC and 1 year later. Matched samples were compared using Wilcoxon's signed rank test. Patients' NRS scores significantly decreased from 7.64 to 5.54 after inclusion in MDC (*p* < 0.001). A significant decrease in clinic visits (*p* < 0.001) and healthcare utilization (*p* < 0.05) was also observed. Opioid and non-opioid prescriptions did not change significantly (*p* = 0.43). 83% of providers agreed that MDC improved patient care. While previous studies have shown the effect of multi-disciplinary care, we show notable improvements with a team established around a once-a-month MDC.

## Introduction

Chronic pain (CP) costs between $USD 560–635 billion in health care expenditures, loss of productivity, and disability (1). CP is associated with reduced quality of life and significant healthcare utilization including repeated hospitalizations, emergency department (ED) visits, and physician visits (2, 3). Management of CP include medications, physical therapy, steroid injections, surgery, and neuromodulation (4–6). Opioid use has quadrupled since the mid-1990s, despite their limited efficacy and serious adverse effects in treatment of CP (7). Often however these therapies are provided in series by different providers and a long-term treatment plan is not developed.

Multidisciplinary care teams have been shown to be effective in specialist care settings such as oncology, inflammatory bowel disease, pediatrics, and cardiology (8–11). CP patients undergoing multidisciplinary treatment report greater pain relief with more realistic expectations, adherence to medication plans and follow-up appointments, and better health literacy about pain, as compared to patients treated by a single provider (12–14). In chronic pain, multidisciplinary care has the potential to reduce opioid use and improve patients' quality of life and satisfaction with treatment (9, 15). Multi-disciplinary CP teams may include neurologists, anesthesiologists with specialty training in pain, physical medicine and rehabilitation specialists, physical or occupational therapists, functional neurosurgeons, spine surgeons, social workers, psychologists, nurses and midlevel providers. These teams are often difficult to implement in actuality, and a starting point is setting common goals and intermediary steps. One step is the creation of monthly multidisciplinary conferences (MDC) for the most complex CP patients.

At our tertiary center, CP patients were referred to a newly formed MDC by their treating physician and were discussed by the care team to create an individualized and holistic treatment plan. We examined outcomes 1 year after patients were added for discussion at MDC, including patients' pain relief and healthcare utilization (hospitalizations, clinic visits, emergency department visits, and medication use). Additionally, we surveyed team members' views of the conferences. Findings from this study could encourage the use of a single monthly MDC to facilitate care of complex chronic pain patients as a starting point for multi-disciplinary care.

## Materials and Methods

### Study Design

A multidisciplinary team of physicians, nurse practitioners, physician assistants, registered nurses, and medical assistants in anesthesia, pain neurology, psychology, and neurosurgery (functional and spine specialties) was established at Albany Medical Center. Patients were referred to the MDC by their treating provider after failure of multiple therapies and/or if their diagnosis was complex and complicated by comorbidities. As these conferences were initiated to improve quality of patient care, our Institutional Review Board approved data collection and publication with an appropriate HIPAA waiver.

### Participants

Thirty-two chronic pain patients were discussed in MDC between May 2019 to December 2020 and included in this study. Only patients with at least 1 year follow-up since time of inclusion in MDC were considered. Demographic data were collected including age, sex, primary and comorbid pain diagnoses, past medical and surgical history, and insurance.

### Data Sources

The number of clinical visits (in person and telehealth) to pain providers, hospitalizations, ED visits, and pain medications were collected from patients' medical records for the year preceding enrollment in the conference and 1 year after enrollment. We also calculated total health care utilization (HCU) using our own equation (HCU = number of hospital visits + number of ED visits + number of physician visits) which we developed based on similar studies (16–19). Included visits were specified as being related to pain or a therapy or intervention related to pain. Information regarding neuromodulatory therapy including spinal cord stimulation (SCS), peripheral nerve stimulation (PNS), and intrathecal drug delivery (IDD) was also collected. In addition, numeric rating scale (NRS) scores were captured immediately preceding the conference and 1 year after. For analysis, opioid medications were converted to morphine milligram equivalent (MME) doses using the online CDC MME Calculator (20). The number of medications was used to track non-opioid medication usage. We verified our records with iSTOP, New York State's prescription monitoring program.

Questionnaires were sent to providers participating in the MDCs to evaluate the impact MDC had on patient care. Providers were asked whether they agreed, held a neutral option, or disagreed with the conference's success in simplifying and improving care, providing more comprehensive treatment plans, and improving pain relief, communication between colleagues, fellow teaching, and job satisfaction. The survey was sent three times to improve response rate.

### Statistical Analysis

Baseline statistics were compared using independent sample *t* tests. Healthcare utilization and outcome measures before and after enrollment were assessed using Wilcoxon signed-rank tests. Results were reported as mean ± standard error of the mean (SEM) unless stated otherwise. A *p* < 0.05 was considered statistically significant. Data were analyzed using GraphPad Prism Software Version 7 (La Jolla, CA, USA). Additional figures were generated with MATLAB Release 2018b (Mathworks, Natick, MA, USA).

## Results

### Demographics

A total of 32 chronic pain patients were included in this study. A summary of the demographic data is shown in Table 1. Twenty-two patients (68.75%) identified as female and 10 patients (31.25%) male. The mean age ± SD for all patients was 56 ± 13.90 years. The etiologies of chronic pain included post-laminectomy syndrome (37.5%), pelvic pain (12.5%), trigeminal neuralgia (9.38%), neuropathic pain (15.63%), migraine (6.25%), and post-herpetic neuralgia (6.25%). Other diagnoses include small fiber neuropathy (*n* = 1), continuous CSF leak (*n* = 1), avascular necrosis (*n* = 1), scleroderma (*n* = 1), treatment resistant lumbar radiculopathy (*n* = 1), and complex regional pain syndrome (CRPS; *n* = 1). Five patients (15.63%) had an additional comorbid pain diagnosis of post-laminectomy syndrome. Twenty patients (62.5%) had previously undergone spine surgeries and 16 patients (50%) were treated with neuromodulation. Payer mix included commercial insurance (71.9%), government insurance (25%), and worker's compensation insurance (3.13%).

**Table 1:** Pre-inclusion patient demographics and clinical characteristics.

	***N***	**SEM**	**%**
**Demographic**			
Female	22		(69)
Age (± SD)	56	± 14	
Daily opioid use (MME/day)	30.4	± 13	
Clinic visits (visits/year)	8.0	± 1	
NRS	7.6	± 3	
**Diagnosis**			
*Post-laminectomy syndrome*	12		(38)
*Chronic Pelvic Pain*	4		(13)
*Complex regional pain syndrome*	1		(3)
*Neuropathic pain*	5		(16)
*Migraine*	2		(6)
*Post-herpetic neuralgia*	2		(6)
*Trigeminal neuralgia*	3		(9)
*Other*	3		(9)
Prior surgery	20		(63)
Neuromodulation	16		(50)
ER utilization	7		(22)

### Pain Intensity Outcomes

Wilcoxon Signed-Ranks test indicated post-enrollment NRS scores were significantly lower than pre-enrollment NRS scores (Z = −4.02, *p* < 0.001), such that the mean NRS was 7.64 ± 0.30 before enrollment and 5.54 ± 0.50 after enrollment. Mean difference was 2.11 ± 0.5. Twenty-one patients (65.6%) experienced a decrease in NRS pain scores after enrollment, while seven patients (21.88%) experienced no change. Four patients did not have NRS scores documented.

### Healthcare Utilization Outcomes

**Figure 1:**
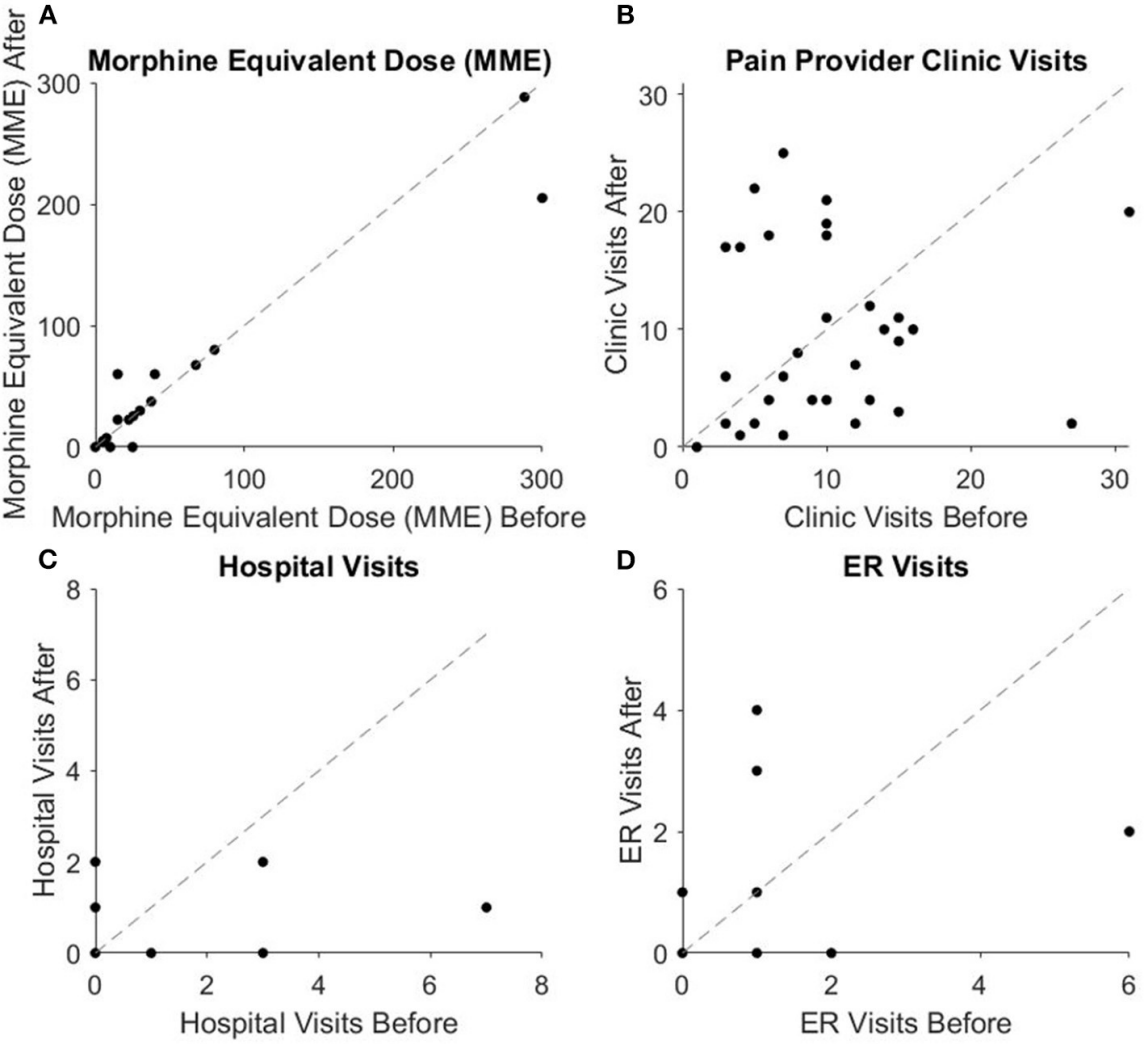
Scatter plots showing qualitative visualization of pain-related data. The dotted line indicates patients who experienced no change with inclusion at multidisciplinary conferences. Data points below and to the right of the line indicate patients with higher **(A)** MME, **(B)** clinic visits, **(C)** hospital visits, and **(D)** ER visits pre-inclusion than post-inclusion, indicating improvement with inclusion at multidisciplinary conference.

To calculate health care utilization scores, we added the number of hospitalizations, the number of outpatient visits (office or clinic visits) and number of ED visits in the year leading up to MDC enrollment and 1-year post-MDC. The number of clinic visits to pain providers and total health-care utilization significantly decreased following inclusion in MDC (Figure 1). Clinic visits to pain providers decreased from 8.03 ± 1.16 to 4.45 ± 0.68 1 year after enrollment (*Z* = −3.21, *p* < 0.001), despite three patients with increased clinic visits after enrollment due to intrathecal pump medication adjustments. Total healthcare utilization decreased significantly as well, from 8.0 ± 1.26 to 4.81 ± 0.74 before and after enrollment respectively (*Z* = −2.64, *p* < 0.05). Twenty-two patients (68.75%) had lower total health care utilization after enrollment while seven patients (21.88%) had higher utilization after enrollment, and three patients (9.38%) had no change.

There were no significant changes in the number of hospitalizations or ED visits related to pain (Figure 1). The mean frequency of hospitalizations related to pain was 0.31 ± 0.15 1-year before enrollment and 0.28 ± 0.11 1-year after enrollment (*W* = 26.5, *p* = 0.96). The average amount of ED visits was 0.41 ± 0.20 pre-enrollment and 0.50 ± 0.17 1-year post-enrollment (*W* = 30.5, *p* = 0.50). Nineteen patients (59.38%) had no ED visits for pain etiologies before or after enrollment. Of the 13 that utilized the ED, eight patients (25%) had more ED visits post-enrollment, four patients (12.5%) had fewer ED visits post-enrollment, and one had the same number of ED visits. Among patients with increased ED visits, eight visits were pain related visits and five visits were due to a pain-related therapy or intervention they were receiving.

### Therapy-Related Outcomes

There was no significant change in medication usage following inclusion in MDC. The mean MME of opioid medications was 30.42 ± 12.72 prior to enrollment and 28.59 ± 11.05 1-year after enrollment (Figure 1) (*W* = 12, *p* = 0.90). MME remained the same for 25 patients (78.13%), decreased for four (12.5%) by an average of 32.81 ± 21.30 MME, and increased for three (9.38%) by an average of 24.17 ± 11.02 MME. On average, patients took 2.03 ± 0.25 non-opioid pain medications 1-year before enrollment and 2.06 ± 0.28 medications after enrollment (*p* = 0.43). Eleven patients (34.38%) used less non-opioid pain medication 1-year after enrollment, while nine patients (28.13%) used more. Medications taken by each patient along with dosage and MME are listed in Table 2.

**Table 2:** Medications and interventions.

**Pt**	**Non-opioid (dose/day)**		**Opioid**			
	**Baseline**	**1 year**	**Baseline**	MME/day^*^	**1 year**	MME/day^*^
1	Duloxetine (120 mg) Gabapentin (2,400 mg)	Duloxetine (120 mg)	+Hydromorphone pump (2.5 mg/day)	0	+Hydromorphone pump (1.25 mg/day)	0
2	Ibuprofen (1,200 mg) + Botox injections	NA + balloon rhizotomy	Oxycodone 15 TID	67.5	Oxycodone 15 TID	67.5
3	Viscous lidocaine (40 ml, PRN)	Viscous lidocaine (40 ml, PRN)	NA	0	NA	0
		Ketamine powder (5 mg)				
	+ Balloon rhizotomy + Botox injections	+Botox injections				
4	Sumatriptan (6 mg)	Sumatriptan (6 mg)	NA	0	NA	0
	Venlafaxine (37.5 mg)	Venlafaxine (37.5 mg)				
	Eptinezumab (100 mg)	Eptinezumab (300 mg)				
5	NA	Gabapentin (600 mg)	NA	0	0	0
		+Ziconotide pump (3.1 mcg/day)				
6	Pregabalin (150 mg)	Escitalopram (20 mg)	NA	0	NA	0
	Topiramate (200 mg)	Pregabalin (150 mg)				
	Trazodone (25 mg)	Topiramate (200 mg)				
	SCS (no longer using)					
7	Pregabalin (600 mg)	Pregabalin (600 mg)	NA	0	NA	0
	Nortriptylene (25 mg)					
8	Diclofenac (50 mg)	Lasmiditan (50 mg)	Oxycodone/APAP 5/325 mg q6 PRN, Butalbital-APAP-Caffeine with codeine (30 mg q6 PRN)	25.5	Hydrocodone/APAP 7.5/325 mg q6 PRN, Butalbital-APAP-Caffeine with codeine (30 mg q6 PRN)	25.5
	Ubrogepant (300 mg)	Cyclobenzaprine (10 mg)				
	Cyclobenzaprine (10 mg)	Sertraline (100 mg)				
	Sertraline (100 mg)					
	+PNS occipital nerve	+PNS occipital nerve				
9	Divalproex (1,000 mg)	Divalproex (1,000 mg)	NA	0	NA	0
	Rizatriptan (10 mg)	Rizatriptan (10 mg)				
	Aspirin (81 mg as an abortive)	Aspirin (81 mg as an abortive)				
	Butalbital-APAP-Caffeine (50–300–40 mg) PRN	Butalbital-APAP-Caffeine (50–300–40 mg) PRN (B)				
10	Fremanezumab (255 mg/month)	Fremanezumab (255 mg/month)	+Ziconotide pump (1.8 mcg/day)	0	+ Pump with Ziconotide (1 mcg/day) and hydromorphone (1 mg/day)	0
	Topiramate (100 mg)	Topiramate (100 mg)				
	Baclofen (10 mg PRN)	Baclofen (10 mg PRN)				
	Sertraline (100 mg)	Sertraline (100 mg)				
11	Gabapentin (300 mg) Escitalopram (5 mg)	NA	NA	0	NA	0
	+DRG stimulation	+DRG stimulation				
12	Amitriptyline (10 mg)	Amitriptyline (10 mg)	Hydrocodone/APAP 5/325 mg daily	5	Hydrocodone/APAP 7.5/325 mg half tab qHS	3.75
		Viscous lidocaine 2% (100 ml, apply PRN)				
13	Bupropion (300 mg)	Bupropion (300 m)	Methadone	80	Methadone	80
	Cyclobenzaprine (15 mg)	Cyclobenzaprine (15 mg)				
	Diclofenac (100 mg)	Diclofenac (100 mg)				
	Pregabalin (500 mg)	Pregabalin (500 mg)				
	+SCS	+SCS, DRG without success				
14	CBD oil	Lidocaine 5% patches (2 patches PRN)	Hydrocodone-acetaminophen 7.5–325 mg qd	7.5	Hydrocodone-acetaminophen 7.5–325 mg qd	7.5
	Duloxetine (60 mg)					
	Naratriptan (2.5 mg PRN)					
15	Ibuprofen (200 mg PRN)		NA	0	NA	0
	SCS (unable to place)					
16	Gabapentin (1,200 mg)	Gabapentin (1,200 mg)	Hydrocodone/APAP 10/325 mg q8 PRN	30	Hydrocodone/APAP 10/325 mg q8 PRN	30
	Alprazolam (0.25)	Alprazolam (0.25)				
	+SCS	+Ziconotide pump (2.7 mcg/day)				
17	Diclofenac 1% gel (PRN)	Diclofenac 1% gel (PRN)	Morphine-sulfate IR	37.5	Morphine-sulfate IR	37.5
18	Cyclobenzaprine (30 mg)	Cyclobenzaprine (30 mg)	Hydrocodone/APA 5/325 q4–6 h	25	NA	0
	Duloxetine (30 mg)	+ DRG SCS				
	+SCS (removed)					
19	Cyclobenzaprine (30 mg)	Nortriptyline (10 mg)	NA	0	NA	0
	Venlafaxine (450 mg)	Tizanidine (12 mg)				
	Lorazepam (4 mg)	Venlafaxine (450 mg)				
		Lorazepam (4 mg)				
	+SCS	+SCS				
20	Alprazolam (1.5 mg)	Escitalopram (40 mg)	Tramadol (150 mg)	15	Hydrocodone/APAP (7.5 mg TID)	22.5
		Alprazolam (1.5 mg)				
		+Ziconotide pump (1.5 mcg/day)				
21	NA +Thoracic SCS +Cervical SCS (removed)	Cyclobenzaprine (10 mg) +Thoracic SCS	Methadone (40 mg) Oxycodone (40 mg)	300	Oxycodone (30 mg) Tapentadol (400 mg)	205
22	Gabapentin (200 mg) Ibuprofen (200 mg PRN)	Ibuprofen (200 mg PRN)	Hydrocodone/APAP (7.5 mg TID)	22.5	Hydrocodone/APAP (7.5 mg TID)	22.5
	Sertraline (50 mg)	Sertraline (50 mg)				
23	Celecoxib (200 mg)	Celecoxib (200 mg)	Tramadol 50 mg BID	10	NA	0
	Duloxetine (60 mg)	Duloxetine (60 mg)				
	Gabapentin (200 mg)	Gabapentin (300 mg)				
		+Ziconotide pump (6.9mcg/day)				
24	Gabapentin (2,700 mg)	Gabapentin (2,700 mg)	Hydromorphone (12 mg)	288	Hydromorphone (12 mg)	288
	Hydromorphone pump (previous treatment)		Fentanyl patch (100 mcg/h)		Fentanyl patch (100 mcg/h)	
25	Butalbital-APAP-Caffeine (50-300-40 mg)	Butalbital-APAP-Caffeine (50-300-40 mg)	Oxycodone (10 mg)	15	Oxycodone (40 mg)	60
	CBD oil	CBD oil				
	Duloxetine (60 mg)	Cyclobenzaprine (15 mg)				
		Duloxetine (60 mg)				
		Lidocaine 5% patch (1 patch PRN)				
		Medical marijuana				
	+SCS	+SCS				
26	NA	Medical marijuana	NA	0	NA	0
		+ Ziconotide pump (placement and removal)				
27	Baclofen (60 mg)	Nortriptyline (100 mg)	NA	0	NA	0
	Cyclobenzaprine (30 mg)	Venlafaxine (150 mg)				
	Divalproex (1,000 mg)	Naltrexone (4.5 mg)				
	Venlafaxine (150 mg)					
	Naltrexone (4.5 mg)					
28	Clonazepam (1 mg)	Clonazepam (1 mg)	NA	0	NA	0
		Pregabalin (300 mg)				
29	Meloxicam (15 mg) + Ziconotide pump (1.3 mcg/day)	Cyclobenzaprine (10 mg) Meloxicam (15 mg) Mirtazapine (22.5 mg) Quetiapine (25 mg) Rizatriptan (5 mg)	Methadone (10 mg)	40	Methadone (15 mg)	60
30	NA	NA	NA	0	NA	0
	+SCS	+SCS				
31	Cyclobenzaprine (30 mg)	Galcanezumab (120 mg/mo) Cyclobenzaprine (30 mg)	Tramadol 50 mg	5	Tramadol 50 mg	5
32	Medical marijuana	Medical marijuana	NA	0	NA	0
	Tizanidine (8 mg PRN)	Tizanidine (8 mg PRN)				
	Mirtazapine (7.5 mg)	Mirtazapine (7.5 mg)				
	Sertraline (100 mg)	Sertraline (100 mg)				
	+PNS brachial plexus	+PNS brachial plexus				

*This includes all pain-related medication and routes, including oral, transdermal, or infusions. Neuromodulation interventions are listed in the table. NA, not applicable*.

* *MME from IDD are not included in the MME calculations*.

Twenty patients' (62.5%) therapy involved neuromodulation. Ten (31.25%) patients had neuromodulation therapy throughout the study period. Three (12.5%) patients had neuromodulation added to their therapy regimen. Five patients were referred for multidisciplinary care as a result of failure of neuromodulation. One patient had an explant and one patient added IDD to existing SCS.

### Provider Perception

**Figure 2:**
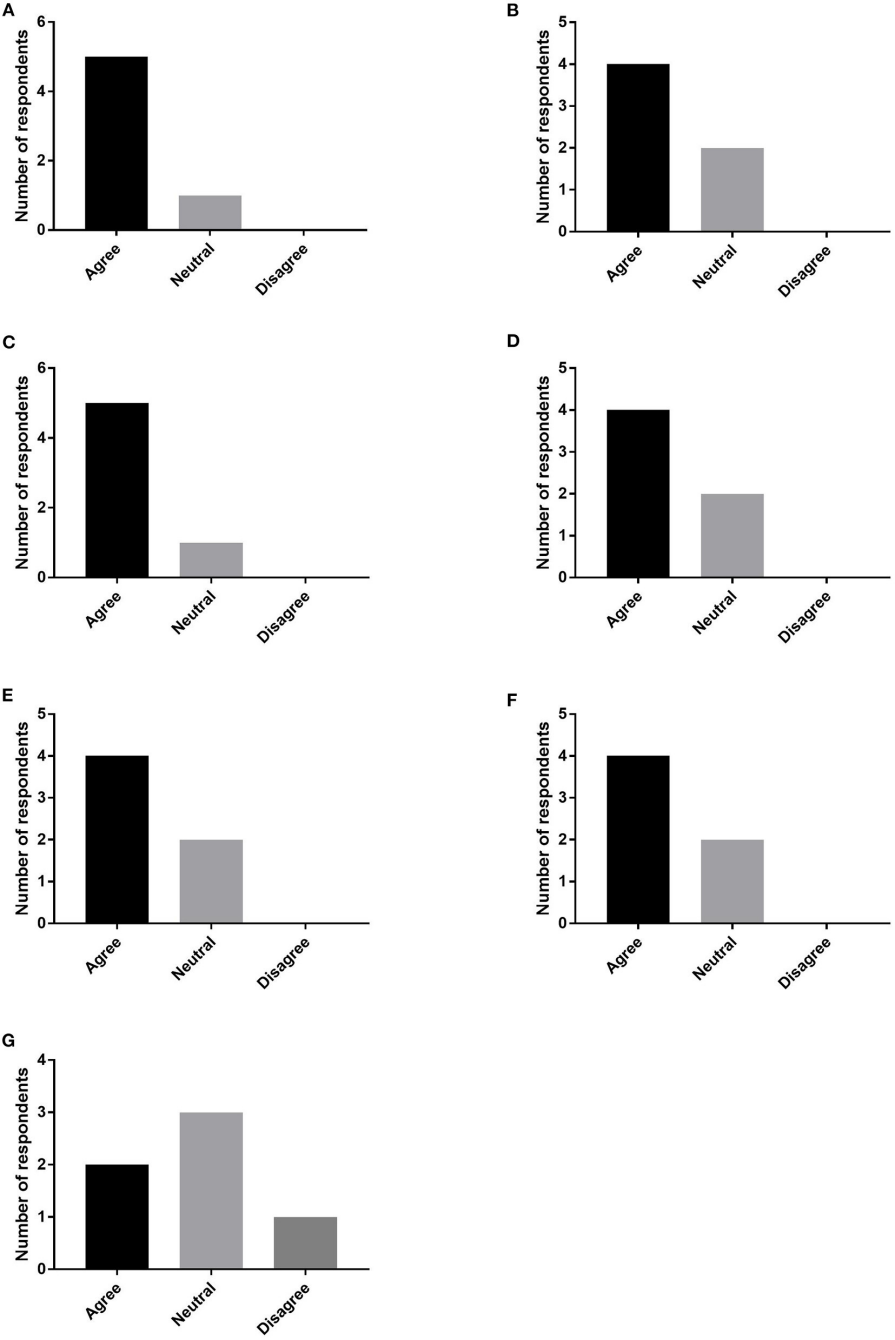
Provider survey responses regarding satisfaction with MDC. Providers indicated whether they agreed, disagreed, or neither agreed or disagreed with the following statements: MDC **(A)** improved patient care, **(B)** streamlined patient care, **(C)** provided a comprehensive treatment plan, **(D)** provided more pain relief, **(E)** improved communication between colleagues, **(F)** improved fellow teaching, and **(G)** improved job satisfaction.

Six of 13 team members responded (46% responder rate). The responders included four physicians (66.67%), one registered nurse (16.67%), and one advanced practice provider (16.67%). Five responders (83.33%) agreed that the conferences improved patient care as a whole and provided patients with more comprehensive treatment plans. Four responders (66.67%) agreed than the conferences provided patients with more pain relief, streamlined patient care, improved communication between colleagues, and improved teaching (Figure 2).

## Discussion

We assessed healthcare utilization, pain outcomes, and medication use for the most complex chronic pain patients before and after inclusion in a newly formed MDC. Only 2% of our total patients were felt to warrant inclusion based on expertise of pain providers on our team. We observed a clear shift in healthcare utilization following inclusion in a multidisciplinary discussion. Patients achieved more pain relief with fewer clinic visits during the year after MDC inclusion. Medication use, hospitalizations and ED visits remained stable. Five of 6 providers felt that MDC improved holistic care for patients.

At the time of inclusion in MDC, our patients had a mean NRS score between 7 and 8, categorized as severe pain, despite having a mean of 8 pain clinic visits, 0.41 emergency visits and 0.31 hospitalizations a year. Minimal clinically important differences (MCID) have been established to accurately determine clinically significant and insignificant improvements in subjective pain rating scales (21, 22). The MCID work suggest that in complex cases, it may not be feasible to decrease pain by 5–6 points due to comorbid pain diagnoses. We observed a 2.1 decrease in NRS in our patients, exceeding the MCID for NRS in complex pain patients of 1.2 (21, 22). The most important aspect of pain management is a realistic expectation for the extent of pain relief. Such reductions in pain may alleviate the burden of frequent clinic visits, and still represent a significant improvement in patients' quality of life.

Clinic visits decreased 60% after inclusion in the MDC and ED and hospital visits remained the same. ED and hospitalization rates were lower than typical in this group of patients (2). We suspect these rates remained low and stable because of the severe exacerbations which are sporadic regardless of whom is treating the patient (23). Generally, chronic pain patients understand the range in which their pain oscillates, so as long as the pain is within that range, they do not seek care. However, when the pain exceeds the normal limit, patients may become concerned and seek the most readily available healthcare option, often the ED.

Our patients' management did include opioids, which is not surprising as a tertiary center. Stringent regulations often prevent patients from receiving opioids, regardless of proof of need and careful observation by professionals (24). Additionally, physicians often turn away patients who are taking prescription opioids, adding further barriers to opioid usage, even when necessary (25). It is worth discussing the two patients who were prescribed doses significantly above recommended dosing levels. One patient had previously been treated with multiple intrathecal pumps but had a number of infections/erosions at outside facilities, ultimately resulting in at least 3 total device removals. Her high opioid dosing regimen has been stable for several years afterwards. MDC did not alter her care which remained stable at 5 clinic visits, 0 hospitalizations, and 0 ED visits in the year after inclusion but did improve her NRS from 6 to 4. She was offered additional neuromodulatory options but declined. Another patient was included after years of multiple provider pain care and shifting regimens including thoracic SCS. After MDC inclusion, his provider visits decreased from 15 to 9, phone calls from 13 to 3, and hospitalizations from 3 to 0; ED increased from 1 to 3. His opioid dose had decreased by the 1-year mark after MDC inclusion, and ultimately decreased further at subsequent time points; his NRS remains stable at 5.

Finally, we show that MDC increased physician perception of patient care, and improved communication, fellow teaching, and job satisfaction. MDCs allows physicians of all backgrounds and specialties to learn from one another, exposing them to different perspectives and integrating expert advice from various specialties (26). Further, team care not only reduces the burden on one provider but allows for collaboration when a provider feels stuck (27). MDC also provides structure to patient care and prevents patients from “falling through the cracks” (28). Additionally, physicians are continuously reminded to review and re-evaluate the treatment course, not just when the patient comes to clinic, to ensure an effective and comprehensive plan is in place (29). Finally, with MDC, patients receive not just one, but a panel of pain specialists to find a comprehensive treatment option which specifically addresses their needs (30).

### Limitations

We acknowledge our study has limitations. First, it is retrospective. Next, we created our own healthcare utilization score equation based on several studies utilizing a similar method (16–19). Next, while we have a number of traditional providers in attendance at our MDC who are well versed in Western medicine, our team did not include alternative therapy such as chiropractic and acupuncture. We did however make recommendations for biofeedback and mindfulness-based interventions and supported use of alternative therapy. Additionally, while the satisfaction survey was distributed several times to improve responder rate, it is possible that the respondents may have only been people who felt strongly about the topic. Future studies will survey a larger cohort of providers to minimize any effects of bias.

## Conclusion

We aimed to evaluate the efficacy of MDC in pain relief and decreased healthcare utilization in the top 2% of complex patients in our practice. While previous studies have shown the effect of multi-disciplinary care, we show notable improvements with a team established around a once-a-month MDC. Our study suggests that the implementation of MDC will not only provide better patient care but will improve provider satisfaction when working with complex pain patients.

## Data Availability Statement

The raw data supporting the conclusions of this article will be made available by the authors, without undue reservation.

## Ethics Statement

The studies involving human participants were reviewed and approved by Albany Medical College Institutional Review Board. Written informed consent for participation was not required for this study in accordance with the national legislation and the institutional requirements.

## Author Contributions

JP created and coordinated the study, provided patient care, directed chart review and analysis, and assisted in writing the manuscript. ZC, GD, OK, RT, and MN also assisted in writing the manuscript. GD and OK composed figures and tables. CA and JP provided patient care and patient data. ZC, GD, OK, and RB performed chart review and statistical analysis. All authors contributed to manuscript editing and approved the final submission.

## Conflict of Interest

JP is a consultant for Boston Scientific, Nevro, Medtronic, Saluda and Abbott and receives grant support from Medtronic, Boston Scientific, Abbott, Nevro, NIH R01CA166379 and NIH U44NS115111. She is the medical advisor for Aim Medical Robotics and Karuna and has stock equity. CA is a consultant for Teva, Lilly, Allergan, Amgen, Novartis, and Flowonix. He provides research support to Teva and Allergan. He has stock in Pfizer. He is part of the Speaker's Bureau for Theranica, Tercera, Teva, Lilly, Allergan, Amgen, Novartis, and Flowonix. GD receives fellowship support from Medtronic, Boston Scientific, and Abbott. The remaining authors declare that the research was conducted in the absence of any commercial or financial relationships that could be construed as a potential conflictof interest.

## Publisher's Note

All claims expressed in this article are solely those of the authors and do not necessarily represent those of their affiliated organizations, or those of the publisher, the editors and the reviewers. Any product that may be evaluated in this article, or claim that may be made by its manufacturer, is not guaranteed or endorsed by the publisher.
